# Tracer‐Based Metabolic NMR‐Based Flux Analysis in a Leukaemia Cell Line

**DOI:** 10.1002/cplu.201500549

**Published:** 2016-03-22

**Authors:** John B. Carrigan, Michelle A. C. Reed, Christian Ludwig, Farhat L. Khanim, Christopher M. Bunce, Ulrich L. Günther

**Affiliations:** ^1^ Institute of Cancer and Genomics SciencesUniversity of BirminghamBirminghamB15 2TTUK; ^2^ Institute of Metabolism and Systems BiologyUniversity of BirminghamBirminghamB15 2TTUK; ^3^ School of BiosciencesUniversity of BirminghamBirminghamB15 2TTUK

**Keywords:** cancer, isotopic tracers, leukaemia, metabolism, NMR spectroscopy

## Abstract

High levels of reactive oxygen species (ROS) have a profound impact on acute myeloid leukaemia cells and can be used to specifically target these cells with novel therapies. We have previously shown how the combination of two redeployed drugs, the contraceptive steroid medroxyprogesterone and the lipid‐regulating drug bezafibrate exert anti‐leukaemic effects by producing ROS. Here we report a ^13^C‐tracer‐based NMR metabolic study to understand how these drugs work in K562 leukaemia cells. Our study shows that [1,2‐^13^C]glucose is incorporated into ribose sugars, indicating activity in oxidative and non‐oxidative pentose phosphate pathways alongside lactate production. There is little label incorporation into the tricarboxylic acid cycle from glucose, but much greater incorporation arises from the use of [3‐^13^C]glutamine. The combined medroxyprogesterone and bezafibrate treatment decreases label incorporation from both glucose and glutamine into α‐ketoglutarate and increased that for succinate, which is consistent with ROS‐mediated conversion of α‐ketoglutarate to succinate. Most interestingly, this combined treatment drastically reduced the production of several pyrimidine synthesis intermediates.

## Introduction

Our previous work^1^, ^2^, ^3^ and that of others^4^ showed that high levels of reactive oxygen species (ROS) have a profound impact on acute myeloid leukaemia (AML) cells, and that susceptibility to ROS represents an Achilles heel that can be used to specifically target AML cells with novel therapies.

We have previously shown the anti‐leukaemic and anti‐lymphoma activity of a combination (denoted BaP) of two redeployed drugs, the contraceptive steroid medroxyprogesterone (MPA) and the lipid‐regulating drug bezafibrate (BEZ).^1^ Consistently associated with BEZ treatment is the rapid and sustained generation of ROS.^1^ We have also shown that BaP treatment has a profound effect on the metabolome of AML cell lines (KG1α, K562, and HL60).^2^ The most pronounced changes were observed for metabolites of the Krebs cycle. HL60 cells showed reduced levels of fumarate compared to succinate, which was attributed to the formation of malonate under BaP treatment, which inhibits succinate dehydrogenase.

Given these findings, and the success of these NMR studies in elucidating mitochondrial insult by ROS, we used a tracer‐based metabolic analysis (metabolic flux analysis) to characterise the effect of this combination of drugs on central carbon metabolism. Tracer‐based metabolic analysis represents a form of targeted metabolomics, in which the distribution of ^13^C from an isotopically labelled metabolic precursor is traced among various metabolites, allowing for a more quantitative interpretation of metabolic changes than is currently afforded by traditional 1D ^1^H spectra. Moreover, NMR spectroscopy yields information on site‐specific label incorporation. Tracer‐based studies enable specific assignments to metabolic pathways, depending on the choice of the isotopically labelled metabolites. NMR spectroscopy and mass spectrometry have been commonly used to determine the incorporation of isotopic precursors such as ^13^C‐labelled forms of glucose and glutamine (reviewed in Ref. 5).

Here we report a tracer‐based metabolic analysis of K562 cells with and without BaP treatment, in which we probe the use of glucose and glutamine in central carbon metabolism. We ascertained the early effects arising from BaP treatment by monitoring label incorporation, as well as alterations to metabolites observed only in natural isotopic abundance. We used [1‐^13^C]glucose and [1,2‐^13^C]glucose as tracers to probe label incorporation in glycolysis and transfer into the Krebs cycle, and [3‐^13^C]glutamine to further probe the Krebs cycle and glutaminolysis. Label incorporation into metabolites was examined using ^1^H–^13^C heteronuclear single quantum coherence (HSQC) NMR spectra. HSQC spectra show a signal per CH group, and in addition, if acquired with sufficient resolution in the incremented ^13^C dimension, show couplings between adjacent carbons. The former data provide direct information about site‐specific label incorporation rates, the latter provides information about label incorporation at adjacent carbon positions.^5^

## Results

HSQC spectra of K562 cell extracts allowed unambiguous identification of approximately 40 metabolites, some of which showed isotope enrichment for cells grown for 24 h with [1,2‐^13^C]glucose or [3‐^13^C]glutamine. Cells were grown with labelled precursors for 24 h, as this is the time frame in which label incorporation can be observed into a wide range of metabolites. Reference spectra from cells grown with glucose and glutamine of natural isotopic abundance were prepared as controls. Although apoptosis or differentiation is usually not evident until about 96 h, our previous work showed a distinct metabolic response after 24 h. Therefore, we also chose a 24 h time frame for the exposure to BEZ and MPA running in parallel with label incorporation. For many of the observed metabolite resonances, corresponding NMR signals could also be observed in unlabelled samples grown with natural isotopic abundance glucose or glutamine.

Here we examine metabolic pathways by using [1‐^13^C]glucose, [1,2‐^13^C]glucose and [3‐^13^C]glutamine to monitor metabolism in K562 cells and determine how it is affected by Bap treatment.

### Glucose mainly feeds the pentose phosphate pathway and glycolysis

**Figure 1:**
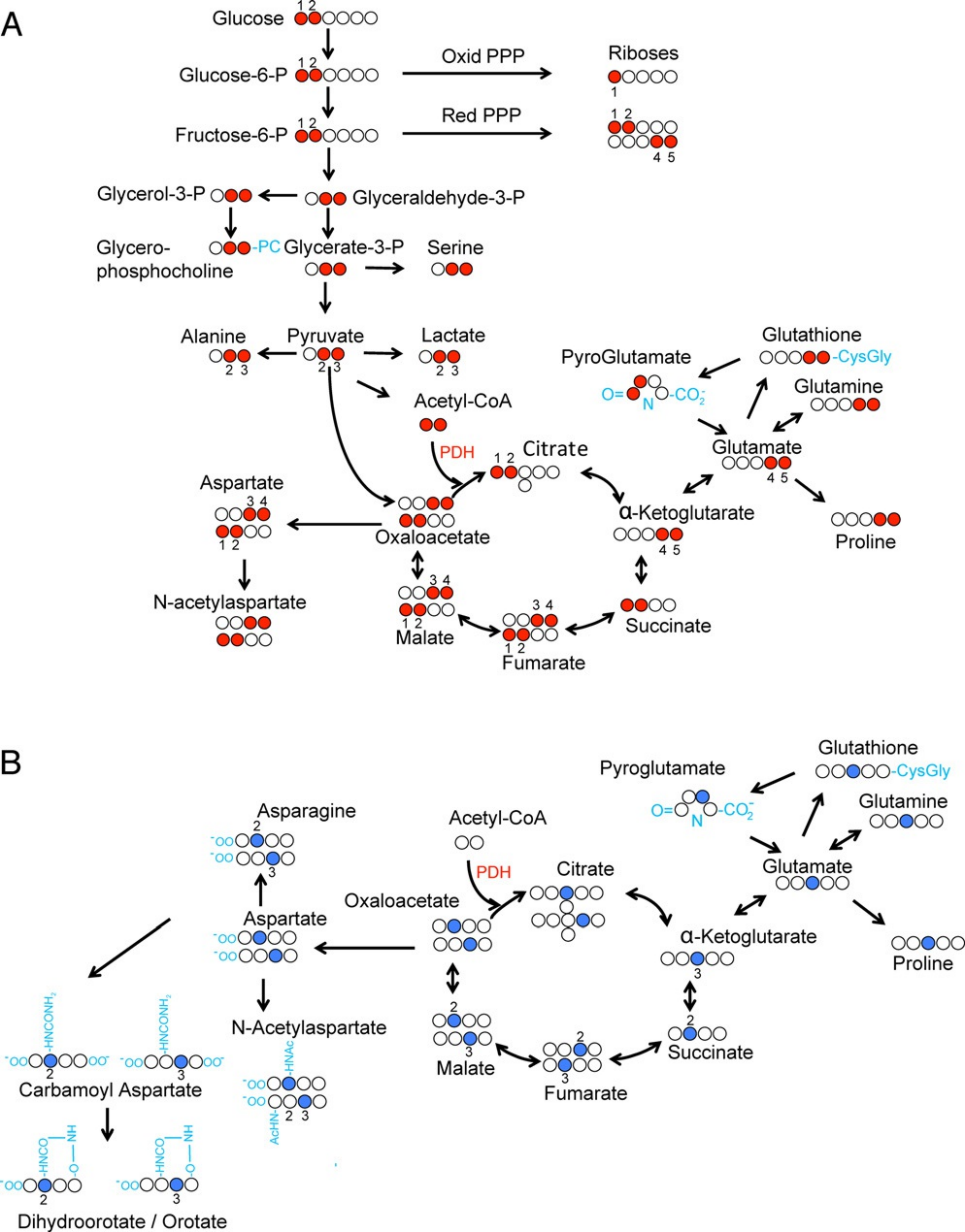
A) Theoretical flow of ^13^C label from [1,2‐^13^C]glucose, assuming PDH‐mediated flow of label into the TCA cycle (red balls indicate labelled positions). B) Theoretical flow of ^13^C label from [3‐^13^C]glutamine (blue balls indicate labelled positions).

**Figure 2:**
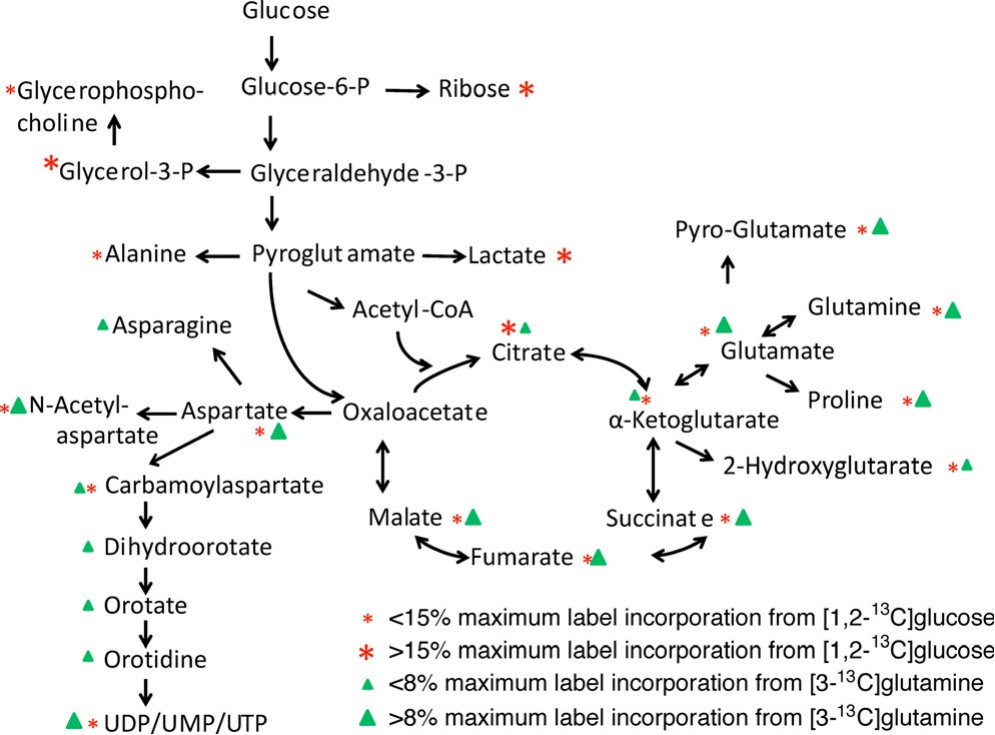
Percentage levels of label incorporation arising from both glucose and glutamine labelling.

**Figure 3:**
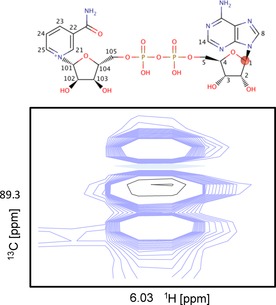
Overlay of the spectral region in which resonances corresponding to the C1 position of NAD^+^ appear. Blue: control sample grown in [1,2‐^13^C]glucose‐supplemented media; black: control sample grown in natural isotopic abundance media.

Figure 1 shows the theoretical labelling patterns in glycolysis and Krebs cycle intermediates arising from feedings cells with [1,2‐^13^C]glucose (Figure 1 A) and [3‐^13^C]glutamine (Figure 1 B). Figure 2 and Table S1 (in the Supporting Information) summarise the experimentally observed incorporation of labels for some key metabolites arising from [1,2‐^13^C]glucose, observed and quantified from K562 cells by HSQC spectra (see Table S1 for further details).

#### Labelling in ribose moieties

The highest isotopic enrichment levels observed were of the ribose sugar resonances, due to pentose phosphate pathway (PPP) activity. Interestingly, labelling of the ribose sugars is indicative of a mix of both oxidative and non‐oxidative PPP activity. In the oxidative PPP, the label from the C1 position of glucose is lost when CO_2_ is released by the action of 6‐phosphogluconate dehydrogenase, yielding ribose sugars labelled exclusively at C1. This results in a single ribose signal for C1 in the HSQC spectrum. In non‐oxidative PPP, the ^13^C1−^13^C2 moiety is retained and gives labelling of C1−C2 and C4−C5 moieties in the resulting ribose sugars. These two ^13^C fragments produce doublets arising from scalar *J*_CC_ couplings observed at C2, C4 and C5.^6^ From C1, three signals arise: the middle singlet signal arises from the oxidative PPP with C2 unlabelled and the outer doublet arises from the non‐oxidative PPP. As an example, Figure 3 shows the well‐resolved adenine ribose C1 signal from nicotine adenine dinucleotide (NAD^+^). The signal arising from the natural abundance sample is very weak (shown in black) relative to the signals measured in the spectrum obtained from the [1,2‐^13^C]glucose‐fed cell extracts (blue). Figure S1 shows all the ^13^C signals for uridine diphosphate (UDP). Here, the C1 regions suffer from substantial signal overlap of the various uridine nucleotide species. However, doublets are clearly observed for C2 and C4, indicative of non‐oxidative PPP activity.

#### Labelling in glycolysis intermediates

Other large enrichments were observed for metabolites produced from pathways branching from glycolysis. For example, glycerol 3‐phosphate and glycerophosphocholine are labelled predominantly at the C2 and C3 positions of the glycerol moiety due to processing from [2,3‐^13^C]glyceraldehyde 3‐phosphate. Likewise, lactate and alanine are labelled at the C2 and C3 positions due to the formation from [2,3‐^13^C]pyruvate.

#### Labelling in Krebs cycle intermediates

Lower levels of label incorporation were observed for metabolites arising from pyruvate entering the TCA cycle, predominately by pyruvate dehydrogenase (PDH)‐catalysed condensation of [1,2‐^13^C]acetyl‐CoA with oxaloacetate. The glutamate labelling pattern is consistent with the incorporation by PDH catalysis of ^13^C into a C4C5 fragment from [1,2‐^13^C]glucose, along with lower label incorporation at the C2 and C3 positions, possibly arising from pyruvate carboxylase activity or further turns of the TCA cycle. The labelling patterns in both glutathione and pyroglutamate in the γ‐glutamyl cycle, and in proline are all consistent with flow of label from glutamate (Figures 1 A and 2).

Similarly, aspartate, produced from the TCA cycle intermediate oxaloacetate, is clearly labelled at the C2 and C3 positions. Interestingly, this label is not transferred into asparagine. However, the label in aspartate is fed into pyrimidine‐base ring synthesis, as shown by the presence of multiplets in the spectra arising from the aspartate‐derived portion of pyrimidine rings (Figure S2).

Four‐carbon TCA cycle intermediates such as succinate, fumarate, malate and aspartate showed labelling of only approximately 5 %, suggesting that glucose is not the primary source of these TCA cycle intermediates. Following this observation, additional experiments were conducted to probe the importance of glutamine as a possible nutrient source for the TCA cycle. Other resonances arising from natural abundance metabolites were also observed in HSQC spectra but are not specifically labelled from glucose: glutamine is the main anaplerotic substrate

Considering that few glucose‐derived ^13^C carbon atoms entered the Krebs cycle we wondered whether glutamine could serve as an anaplerotic substrate in K562 cells. This was investigated using [3‐^13^C]glutamine as a tracer. Indeed, we observed much higher label incorporation in Krebs cycle intermediates compared to that observed if glucose was used as a tracer (Figure 2, Table S1). The most intensely labelled carbon positions in metabolites (Table S1) corresponded to the expected labelling pattern that would arise from the conversion of [3‐^13^C]glutamine to glutamate, catalysed by mitochondrial glutaminase, and subsequently to α‐ketoglutarate (Figure 1 B). The overall label incorporation into the cellular pool of the four‐carbon Krebs cycle intermediates was typically fourfold higher than that derived from [1,2‐^13^C]glucose (Table S1).

Outside of the mitochondria, pyrimidine carboxyltransferase catalyses the addition of aspartate to carbamoyl phosphate as part of the first step in pyrimidine nucleotide synthesis. Perhaps because of the much greater extent of labelling in aspartate, label incorporation was observed in several intermediates in this pathway; namely *N*‐carbamoylaspartate, dihydroorotate, orotate and orotidine, as well as in the pyrimidine rings of various uridine and cytidine nucleotide species.

Curiously, no label incorporation was seen in lactate, alanine or acetyl groups (by observing signals arising from the acetyl groups of *N*‐acetylated amino acids and acetic acid). This contrasts sharply with work by DeBerardinis et al., who found that 60 % of glutamine was converted to lactate and alanine in glioblastoma cells, a process that involves malate exiting the TCA cycle and mitochondria with subsequent conversion to pyruvate by a malic enzyme, thereby generating sufficient NADPH to support fatty acid biosynthesis.^7^ Although it is currently not understood what regulates this process, it is increasingly clear that it is different between cancer cell types.

### BaP treatment increases intracellular glutamine and reduces de novo pyrimidine synthesis

As we have shown previously,^1^ BaP treatment causes high levels of ROS, as a consequence of the action of BEZ (see also Figure S3). We observed a significant change in label incorporation with BaP treatment for a few metabolites only. The treatment decreased label incorporation from [1,2‐^13^C]glucose into α‐ketoglutarate and increased that for succinate. This is consistent with ROS‐mediated conversion of α‐ketoglutarate to succinate, and with the previous results of Tiziani and co‐workers.^2^ Equally, there was little change in label incorporation observed in spectra from [3‐^13^C]glutamine‐labelled cells upon BaP treatment. Label incorporation into succinate increased and labelling of α‐ketoglutarate decreased, as observed for labelled glucose. Moreover, aspartate and acetyl‐*N*‐aspartate labelling increased slightly upon BaP treatment, whereas the label incorporation in pyrimidine synthesis intermediates *N*‐carbamoylaspartate, dihydroorotate and orotate was significantly reduced. For the latter group, there were no signals present in the reference natural abundance spectra. Therefore, we reasoned that the reference signal had intensity below the noise level in the spectrum. Consequently, we were able to calculate minimum label incorporations by assuming that the reference intensity was the maximum noise in the reference spectrum.

#### Effects of BaP on pyrimidine synthesis

**Figure 4:**
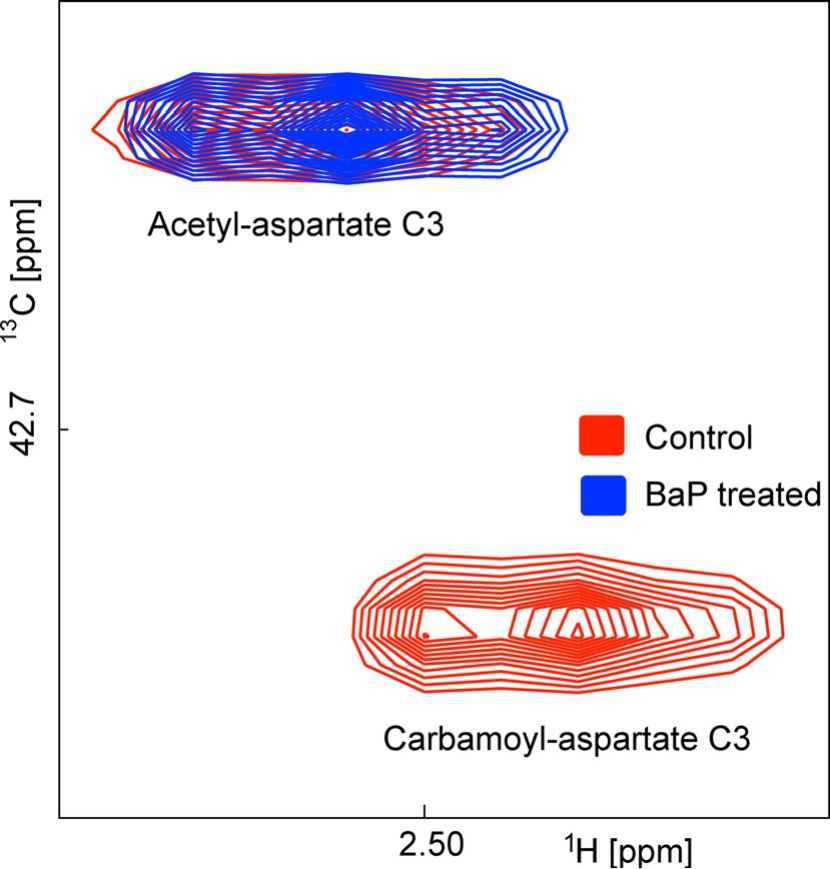
Overlay to compare the effect of BaP treatment on resonances from *N*‐acetylaspartate (top left peak) and *N*‐carbamoylaspartate (bottom right peak). Red: control sample grown in [3‐^13^C]glutamine; blue: BaP‐treated sample.

Figure 4 shows a region of the HSQC spectrum containing the C3 cross peaks of *N*‐acetylaspartate and *N*‐carbamoylaspartate, clearly illustrating their very different responses to BaP treatment. Dihydroorotate and orotate signals, both downstream metabolites in the pyrimidine synthesis pathway are also clearly diminished. However, it remains enigmatic why pyrimidine nucleotides show only a minimal, if any, reduction in label incorporation (see Table 1, [3‐^13^C]glutamine section, C11, C12). To resolve this puzzle we also measured the 1D spectra of unlabelled samples (Figure S4). These showed a change in the balance between UDP‐*N*‐acetylglucosamine and other UDP‐containing species. Whereas just levels of UDP‐glucose, UDP‐glucuronate and UDP‐galactose were clearly reduced with BaP treatment, UDP‐*N*‐acetylglucosamine increased.

**Table 1:** Natural abundance levels of γ‐glutamyl cycle metabolites from HSQC spectra.

	Myo C1/C3*	Gln C_4_	Glu C_4_	GSH C_4_	Pyroglu C_4_
Control^[a]^	1.25×10^7^	1.31×10^6^	1.95×10^7^	4.15×10^6^	5.53×10^5^
Bap	1.09×10^7^	3.39×10^6^	2.08×10^7^	3.19×10^6^	9.1×10^5^
Control‐scaled intensity^[b]^	1.000	0.105	1.560	0.332	0.044
BaP‐scaled intensity^[b]^	1.000	0.311	1.908	0.293	0.083
BaP/control ratio	1.00	2.96	1.22	0.88	1.89

[a] Arbitrary intensity values from original data. [b] *myo*‐Inositol C1 and C3 were used as references, that is, it was assumed that their levels were not affected by BaP treatment. Scaled intensity=metabolite intensity/*myo*‐inositol intensity. Glu, glutamate; Gln, glutamine; GSH, glutathione; Myo, *myo*‐inositol; Pyroglu, pyroglutamate.

#### Effects of BaP on the γ‐glutamyl cycle

The levels of metabolites from the γ‐glutamyl cycle were also perturbed by BaP treatment (Figure S5). ^1^H 1D spectra of unlabelled samples showed that glutamate and particularly glutamine both increased in levels on treatment with BaP (data not shown). Analysis of peak intensities in HSQC spectra derived from cell extracts grown in unlabelled media showed that BaP treatment greatly increased the amounts of glutamine and pyroglutamate, somewhat increased glutamate and slightly decreased glutathione levels (Table 1). Overall this might suggest that cells respond to high concentrations of ROS by increasing their glutamine uptake.

In spectra arising from [3‐^13^C]glutamine‐treated cells, label incorporation at C3 can be assessed from the ratio of the signals of *J*_CC_‐coupled doublet versus the singlet C4. This overcomes the problem that C3 signals of glutamine and glutathione are significantly overlapped. It is important to note that the relative intensities of peaks in the glutathione multiplet did not change significantly with BaP treatment (Table 2), demonstrating no change in label incorporation.

**Table 2:** Labelling in the γ‐glutamyl cycle.

Metabolite	Peak heights, control ×10^6^	Peak heights, BaP ×10^6^
Glutamate C4^[a]^	doublet: 6.22+4.67	doublet: 8.00+5.94
	singlet: 8.24	singlet: 12.9
	ratio: 1.3	ratio: 1.1
		
Glutathione C4^[a]^	doublet: 1.23+1.16	doublet: 1.01+0.92
	singlet: 2.2	singlet: 2.26
	ratio: 1.1	ratio: 0.9
		
Pyroglutamate C3^[b]^	4.55, 3.85	14.7, 15.9

[a] ^13^C4 signal intensities for doublet (arising from coupling to ^13^C3) and singlet (with ^12^C3). [b] Directly observed C3 signal intensities for H_a_ and H_b_.

Label incorporation into glutamate was also not significantly affected by BaP treatment. By contrast, label incorporation from [3‐^13^C]glutamine into pyroglutamate increased threefold upon BaP treatment (Table 2). The increase of pyroglutamate labelling is consistent with cyclisation of glutamate or glutamine to pyroglutamate mediated by ROS.

## Discussion

Overall, the NMR analysis of label incorporation into metabolites arising from labelled glucose and glutamine precursors shows the significant potential of this approach for studying metabolic mechanisms in human cell lines. Although this has originally been recognised by Shulman and Ugurbyl and co‐workers^8^, ^9^, ^10^ and later by Szyperski, Bailey, and Wüthrich,^11^, ^12^ the method of choice for most subsequent studies has been mass spectrometry, probably owing to its much higher sensitivity. However, the limitation of mass spectrometry is that it provides less information from mass increments, whereas NMR can in principle yield additional information for site‐specific label incorporation. If [1,2‐^13^C]glucose is used, further information arises from the ^1^ *J*_CC_ scalar coupling between adjacent carbons, especially as the C1 atom in glucose is abstracted in the entry step to the oxidative PPP, but not for the non‐oxidative PPP branch. In some cases, this is also useful to determine label incorporations into carbon atoms that cannot be easily resolved owing to overlap if an adjacent CH can be observed as a reporter. This has been shown for glutamine‐related metabolites.

HSQC spectra show a larger number of metabolites than directly observed 1D ^13^C spectra, owing to its much higher sensitivity. This is of course limited to carbon atoms with an attached proton, although label incorporation into CO and COO groups can often be inferred from the signals of neighbouring CHs. In order to resolve *J*_CC_ couplings, at least 4096 increments need to be acquired, resulting in an overall acquisition time of more than 4 h for two increments with a recycle time of 2 s.

### K562 central carbon metabolism

This analysis provides new insights into the metabolism of K562 leukaemic cells. This cell line was derived from a BCR–ABL translocation‐positive blast‐crisis chronic myeloid leukaemia and is an established cell‐line model for leukaemia. Our data demonstrates that large amounts of glucose are funnelled into the PPP, as observed by ^13^C labelling of ribose. The observed labelling pattern is indicative of a mix of oxidative and non‐oxidative PPPs, as evidenced by C1–C2 and C4–C5 couplings. This was further supported by label incorporation into various ribose sugars arising from [1‐^13^C]glucose, which is only possible by the non‐oxidative pathway, for example, 36 % label incorporation into C1 of adenosine diphosphate.

Further label incorporation was observed in other glycolysis‐related metabolites such as glycerol 3‐phosphate and lactate, although levels were lower than those observed for ribose sugars. In the Krebs cycle, label incorporations in glutamate, succinate, fumarate and malate were all low (≈5 %) from glucose and larger (13–50 %) from glutamine. This is in accordance with a strong Warburg effect, by which lactate is produced and entry of pyruvate into the Krebs cycle is blocked. Large amounts of glucose‐derived label diverted into the PPP have previously been observed for cancer cells.^13^ The high levels of label incorporation from glutamine indicate highly active glutaminolysis.

In K562 cells, glutamine‐derived label was not observed in lactate or alanine. This is in sharp contrast to the work of DeBerardinis et al.,^6^ who found that 60 % of glutamine converted to lactate in glioblastoma cells. Neither did we observe label incorporation into asparagine, although aspartate is labelled from glucose and, to a lesser degree, from glutamine.

### Effects of BaP treatment

Tracer‐based metabolic analysis of BaP‐treated K562 cells confirmed that α‐ketoglutarate levels are reduced, whereas succinate was increased after a 24 h treatment with BaP. Importantly, BaP treatment reduced label incorporation into pyrimidine synthesis intermediates, especially those of *N*‐carbamoylaspartate, orotic acid and dihydroorotic acid. The de novo synthesis of pyrimidines is crucially important for the cell and its perturbation is likely to have huge implications outside of the immediate synthesis pathway as highlighted in a recent study by He et al. in 2014.^14^ After assembly from bicarbonate, glutamine and adenosine triphosphate, uridine and cytidine nucleotides are fuels for the synthesis of RNA, DNA, phospholipids, UDP sugars and glycogen. In this study, we have for the first time tracked all stages of this process through the labelling of aspartate from our [3‐^13^C]glutamine source. It remains puzzling why label incorporation into the pyrimidine end product is always high and not significantly affected by BaP treatment. One possible explanation is that the pyrimidine pool builds up prior to the inhibition of pyrimidine synthesis or that the pyrimidine salvage pathway becomes more active to maintain these pools.

BaP treatment causes an unexpected shift in the balance of different UDP species, showing decreases in the amounts of UDP‐glucose, UDP‐glucuronate and UDP‐galactose and an increase for UDP‐*N*‐acetylglucosamine. This is apparent from the ribose signals of UDP compounds in 1D spectra of unlabelled samples, whereas the pyrimidine signals of individual UDP species are not separately resolved.

## Conclusion

The data presented in this study highlights the potential of isotopic tracer‐based profiling in understanding cancer cell metabolism and any subsequent metabolic changes arising from treatment. K562 cells clearly display high glycolytic activity, although this is not linked to the Krebs cycle, but rather towards the production of ribose sugars in the PPP and lactate. Moreover, we have observed glutamine as an anaplerotic source for the Krebs cycle. Our analysis also shows the effects of BaP treatment, specifically changes in succinate and α‐ketoglutarate levels, and a reduction of pyrimidine synthesis intermediates. Metabolic changes observed in AML cell lines in response to BaP treatment reflect the downstream effect of ROS as previously reported.

## Experimental Section

### Metabolic labelling and NMR sample preparation

K562 cells were maintained in an exponential phase (2.5–10×10^5^ cells mL^−1^) in RPMI‐1640 medium supplemented with heat‐inactivated 10 % FBS and penicillin/streptomycin (Gibco, Life Technologies Ltd, Paisley, UK) at 37 °C and 5 % CO_2_ in a humidified incubator. A sufficient number of cells, 5×10^7^ exponentially growing K562 cells per control or treatment, were harvested by centrifugation (800 *g*, 5 min).

For tracer‐based metabolic analysis, standard media with natural isotopic abundance precursors or 100 % labelled media were used. Media containing 100 % ^13^C‐labelled precursor was prepared by adding [1,2‐^13^C]glucose (2 g l ^−1^, Isotec; Sigma–Aldrich) to glucose‐free RPMI‐1640 (Gibco) or [3‐^13^C]glutamine (300 mg l ^−1^, Isotec; Sigma–Aldrich) to glutamine‐free RPMI‐1640 (Gibco). BEZ (0.5 mm) and MPA (5 μm; Sigma–Aldrich), or the equivalent concentrations of DMSO and ethanol solvent controls, were added to media. Cell pellets were resuspended in the relevant media and incubated for 24 hrs with labelled precursors and/or BaP at 37 °C and 5 % CO_2_ in a humidified incubator.

Cells were harvested by centrifugation (800 *g*, 10 min) and the pellets were washed with PBS and transferred to 1.8 mL glass vials in PBS (1 mL). Cells were centrifuged (4000 *g*, 4 °C, 1 min), then the PBS was removed and the cell pellet resuspended in methanol (400 μL) that had been pre‐chilled over dry ice, thus freezing the samples. Samples were kept on dry ice and then stored at −80 °C. After removal from −80 °C storage, the vials were placed on wet ice and distilled H_2_O (325 μL) and chloroform (400 μL, prechilled on wet ice) were added before vortexing for 30 s followed by incubation on ice for 10 min to allow the phases to separate. Following centrifugation (1500 *g*, 4 °C, 10 min), the polar (upper) fraction was transferred to Eppendorf tubes and dried overnight in a centrifugal vacuum concentrator.

### NMR data acquisition

Polar extracts were resuspended in metabolomics buffer (60 μL; sodium phosphate buffer, 0.5 mm trimethylpropanoic acid, 10 % D_2_O, pH 7) with vortexing, and supernatant (40 μL) was transferred to champagne vials. Supernatant (35 μL) was then transferred to 1.7 mm NMR tubes and kept at 4 °C prior to measurement.

All spectra were acquired at 298 K on a Bruker 600 MHz spectrometer with a TCI 1.7 mm z‐PFG cryogenic probe using a cooled Bruker SampleJet autosampler. In all experiments, the ^1^H carrier was set to the water frequency and the ^1^H 90° pulse was calibrated at a power of 0.326 W.

For the ^1^H 1D spectra, the standard Bruker pulse sequence noesygppr1d for 1D NOESY with water presaturation was used. The key parameters were as follows: spectral width: 12.15 ppm (7288.6 Hz); complex points, TD: 32768; interscan delay, d1: 4 s; short NOE mixing time, d8: 10 ms; number of scans, ns=256; dummy scans, ds=8. The total experiment time was 28 min.

For the ^1^H–^13^C HSQC spectra the pulse sequence used was based on the Bruker standard pulse program hsqcetgpsp, which uses echo/anti‐echo time‐proportional phase incrementation gradient selection, with additional gradient pulses to improve water suppression. Key parameters for the ^1^H observation dimension were: a spectral width of 7812.5 Hz, 2048 complex points in the direct dimension, 4096 increments for the ^13^C indirect dimension with a spectral width of 159.0 ppm. Spectra were acquired with two scans and an interscan delay of 1.5 s, giving a total experiment time of approximately 4 h. All spectra were processed using NMRLab^15^ in MATLAB. Cosine‐squared window functions were applied to both dimensions.

### Analysis of 2D spectra

Peaks were picked in a semi‐automated manner using MetaboLab.^16^ To calculate percentage label incorporations, the cross peaks in labelled spectra (spectra from cell extracts grown in labelled media) and reference spectra (spectra from cell extracts grown in natural isotopic abundance media) were compared. The ^13^C isotope constitutes about 1 % of naturally occurring carbon. Therefore, for more concentrated metabolites, cross peaks could be observed in the reference spectra. Peak intensities in control and reference spectra were used to calculate the percentage incorporation of ^13^C labels into particular carbon atoms of a given metabolite following the equation % incorporation=100 *N*/(*D S*) where *N* is the intensity of the selected peak of the metabolite in labelled media, *D* is the intensity of the selected peak of the metabolite in the control spectrum, and *S* is the mean of a scale factor. The scale factor=*N*_r_/*D*_r_, where *N*_r_ is intensity of peak *i* from a reference metabolite in the numerator spectrum and *D*_r_ is the intensity of peak *i* from a reference metabolite in the denominator spectrum. The reference metabolite was chosen because it was one of a group of metabolites that did not change in intensity significantly between treated and untreated spectra or between spectra for enriched and natural abundance media. Results were similar using valine, leucine or isoleucine as the reference metabolite. Labelling was not considered significantly changed unless BaP treatment changed percentage label incorporation by at least a factor of 2.

In some spectra, peaks were not observed owing to their low intensity. In such cases, the peak intensity was set to the estimated noise level in that spectrum. That noise level is evaluated by searching for the maximum of the absolute value of the intensity seen in a region devoid of real signals. When calculating percentage incorporations of ^13^C, it is the ^12^C reference spectrum peak intensity that might be missing. Substituting the dummy peak intensity will cause a tendency of the percentage incorporation of ^13^C to be (conservatively) underestimated“.

## Supporting information

As a service to our authors and readers, this journal provides supporting information supplied by the authors. Such materials are peer reviewed and may be re‐organized for online delivery, but are not copy‐edited or typeset. Technical support issues arising from supporting information (other than missing files) should be addressed to the authors.

Supplementary
