# Supplementary material for: Tracer‐Based Metabolic NMR‐Based Flux Analysis in a Leukaemia Cell Line
Source: Chempluschem. 2016 Mar 22;81(5):453–9. doi: 10.1002/cplu.201500549 (PMC4916734; doi:10.1002/cplu.201500549)
Supplement: Supplementary file 1 — Supplementary [file CPLU-81-453-s001.pdf]

CHEMPLUSCHEM

Supporting Information

Tracer-Based Metabolic NMR-Based Flux Analysis in a Leukaemia Cell Line

John B. Carrigan+,[a] Michelle A. C. Reed+,[a] Christian Ludwig,[b] Farhat L. Khanim,[c] Christopher M. Bunce+,[c] and Ulrich L. Günther+*[a]

cplu_201500549_sm_miscellaneous_information.pdf

**Table S1: Percentage label incorporations**		
**[3-^{13}C]Glutamine**	Control	Bez+MPA
Glutamate C3 C3	54,44	38,39
Pyroglutamate C3 C3	16,13	41,32
Proline C3	13,15	14,17
α-Ketoglutarate C3*	17	9
Succinate C2	13	35
Malate C2 C3 C3	44,7,23	34,14,23
Fumarate C2*	15	19
Aspartate C2 C3 C3	12,17,14	15,9,18
Carbamoyl aspartate C2, C3*	12,16	2,2
Dihydroorotate*	5,5	1
Orotate*	6	0
UDP C11 C12	16,13	13,13
Malonate C2	0	2
Citrate C2	15,10	10,10
Lactate C2 C3	1,1	1,1
Alanine C2 C3	1,1	1,1
**[1,2-^{13}C]glucose**		
ADP C1	53	30
Glycerol-3-phosphate C2 C3 C3	28,23,22	34,20,14
Glycerophosphocholine C2 C3 C3	8,9,6	11,12,10
Alanine C2 C3	4,5	9,10
Lactate C3	45	39
Citrate C2	23,19	10,10
Glutamate C2 C3 C3 C4	2,5,4,6	3,4,4,7
Pyroglutamate C2 C3 C3 C4	2,1,2,4	2,1,2,4
Proline C2 C3 C3 C4 C5 C5	3,4,5,6,8,7	3,4,5,6,6,6
α-Ketoglutarate	12	2
Succinate C2	6	13
Malate C2 C3 C3	7,4,4	5,3,5
Fumarate C2*	3	3
Aspartate C2 C3 C3	1,4,1	3,4,5
UDP C11 C12	5,7	5,7
Malonate C2	0	17
**[1-13C]glucose**		
ADP C1	36	19
Glycerol-3-phosphate C2 C3 C3	1,12,13	1,11,15
Glycerophosphocholine C2 C3 C3	1,25,25	1,25,37
Lactate C3	38	32
Alanine C2 C3	1,14	1,15
Citrate C2	33,35	22,18
Glutamate C2 C3 C3 C4	5,6,5,12	5,5,5,13
Pyroglutamate C2 C3 C3 C4	3,1,2,9	3,2,2,6
Proline C2 C3 C3 C4 C5 C5	2,2,3,5,1,1	3,2,2,6,1,1
Succinate C2	4	14
Malate C2 C3 C3	11,4,6	12,8,8
Fumarate C2	1	1
Aspartate C2 C3 C3	11,29,13	13,14,17

UDP C11 C12	6,7	7,6
Malonate C2	0	21

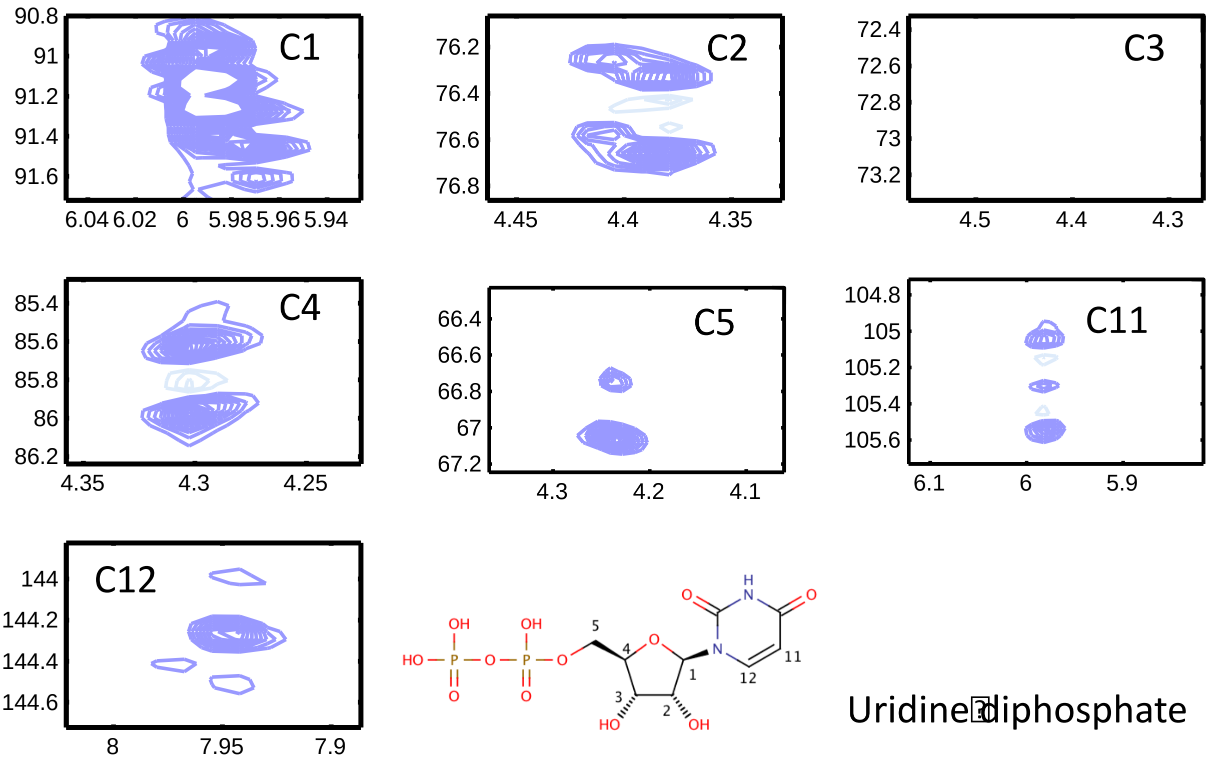

Figure S1: Signals of uridine diphosphate (UDP) in cells labeled with [1,2=^{13}C]glucose. The C1 regions suffer from substantial signal overlap of the various UXP species. However, doublets are clearly observed at C2 and C4, indicative of non-oxidative PPP activity. *C11 and C12 labelling arises from incorporation of labelled aspartate.*

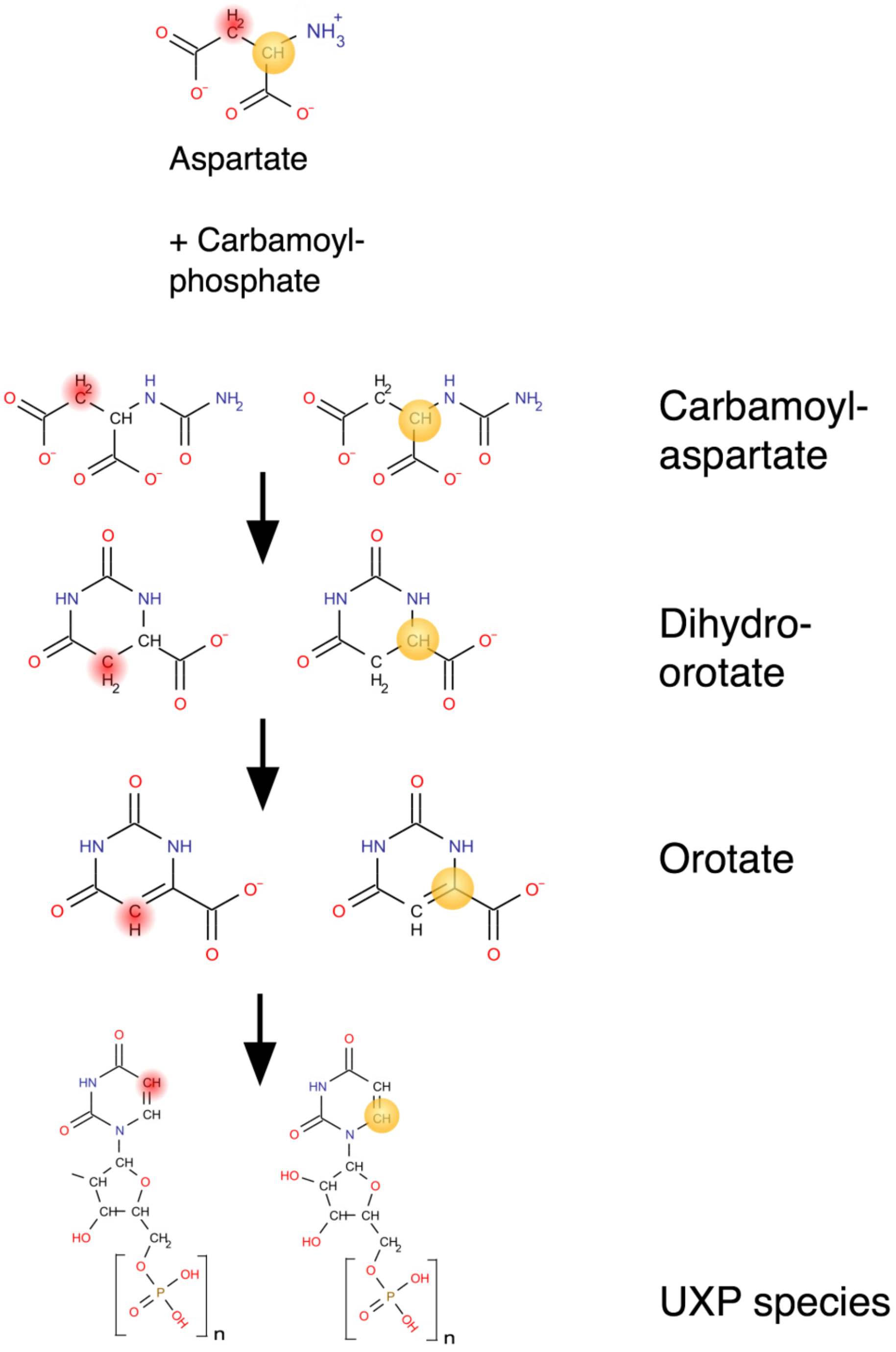

Figure S2: Schematic representation of the flow of carbon atoms in pyrimidine synthesis.

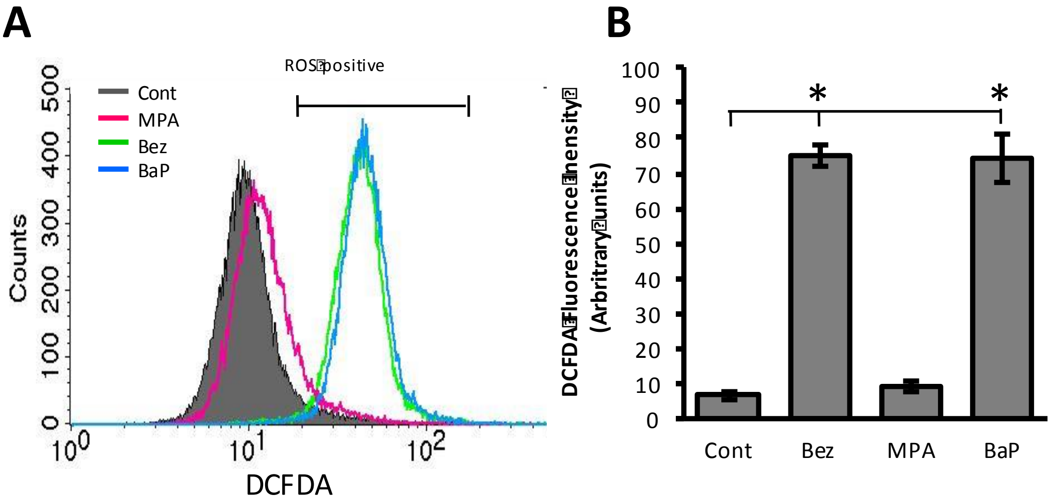

Figure S3: Reactive oxygen species (ROS) and BaP. K562 cells were treated with either solvent control, 0.5mM Bez, 5mM MPA or the combination (BaP) for 14hours. ROS production was assessed by DCFDA staining and analysis by flow cytometry. A) Representative histograms of ROS data. B) Mean data ± SEM of n=4 experiments.

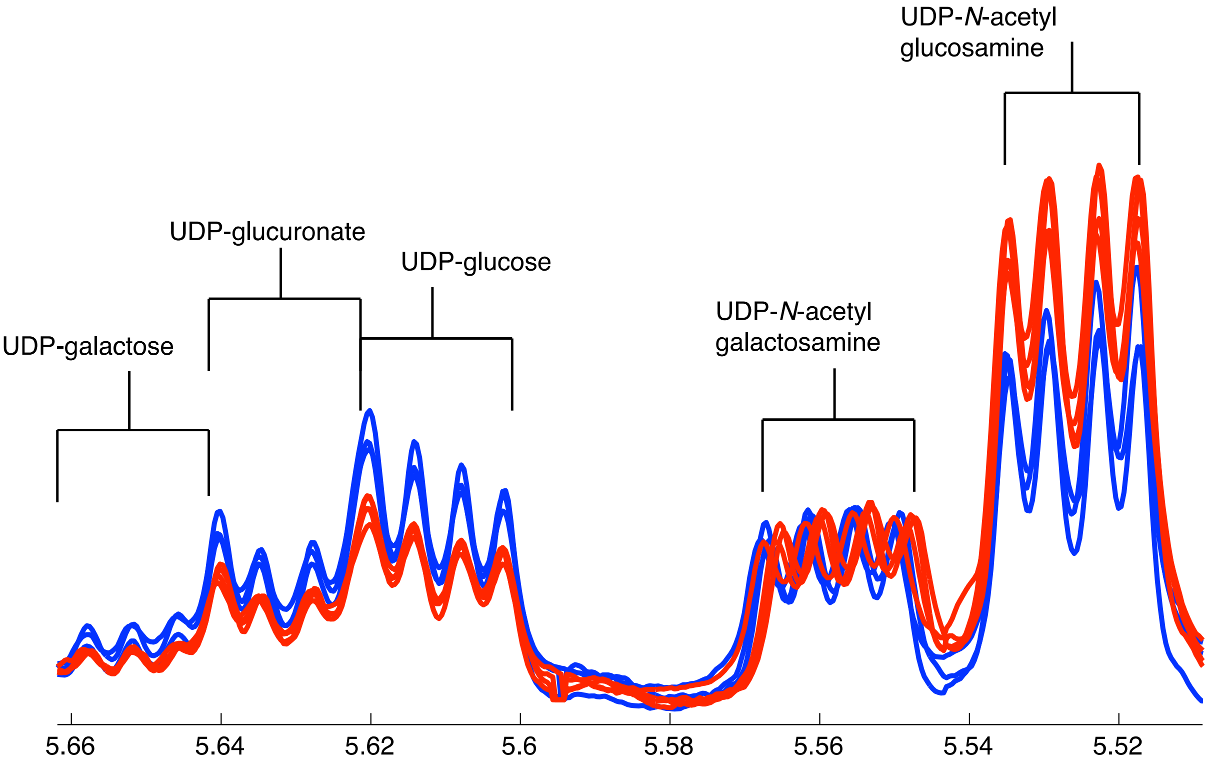

Figure S4: 1D-NMR spectra showing altered production of different UDP containing species.

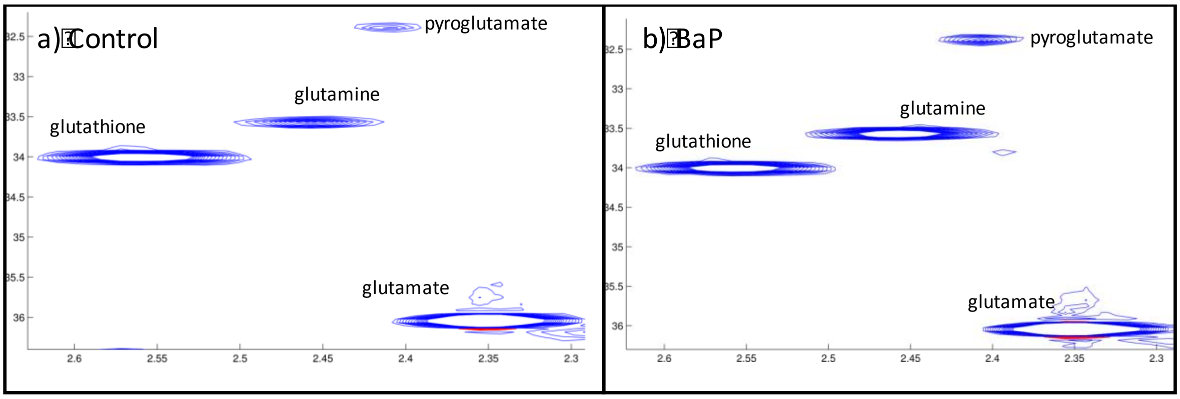

Figure S5: Sections from HSQC spectra of unlabeled samples with (BaP) and without (control) BaP treatment showing that glutamine is raised with BaP.
